# Effect of B-vitamin Supplementation on Stroke: a Meta-Analysis of Randomized Controlled Trials

**DOI:** 10.1371/journal.pone.0081577

**Published:** 2013-11-25

**Authors:** Chi Zhang, Feng-Ling Chi, Tian-Hao Xie, Yu-Hao Zhou

**Affiliations:** 1 Department of Neurosurgery, Shanghai Seventh People’s Hospital, Shanghai, China; 2 Department of Neurosurgery, Changzheng Hospital, Second Military Medical University, Shanghai, China; 3 Department of Rehabilitation Institute, Shanghai Seventh People’s Hospital, Shanghai, China; University of Glasgow, United Kingdom

## Abstract

**Background:**

B vitamins have been extensively used to reduce homocysteine levels; however, it remains uncertain whether B vitamins are associated with a reduced risk of stroke. Our aim was to evaluate the effects of B vitamins on stroke.

**Methodology and Principal findings:**

We systematically searched PubMed, EmBase, and the Cochrane Central Register of Controlled Trials to identify studies for our analysis. Relative risk (RR) was used to measure the effect of B-vitamin supplementation on the risk of stroke. The analysis was further stratified based on factors that could affect the treatment effects. Of the 13,124 identified articles, we included 18 trials reporting data on 57,143 individuals and 2,555 stroke events. B-vitamin supplementation was not associated with a significant reduction in the risk of stroke (RR, 0.91, 95%CI: 0.82–1.01, P = 0.075; RD, -0.003, 95%CI: -0.007–0.001, P = 0.134). Subgroup analyses suggested that B-vitamin supplementation might reduce the risk of stroke if included trials had a man/woman ratio of more than 2 or subjects received dose of folic acid less than 1 mg. Furthermore, in a cumulative meta-analysis for stroke, the originally proposed nonsignificant B-vitamin effect was refuted by the evidence accumulated up to 2006. There is a small effect with borderline statistical significance based on data gathered since 2007.

**Conclusions/Significance:**

Our study indicates that B-vitamin supplementation is not associated with a lower risk of stroke based on relative and absolute measures of association. Subgroup analyses suggested that B-vitamin supplementation can effectively reduce the risk of stroke if included trials had a man/woman ratio of more than 2 or subjects received dose of folic acid less than 1 mg.

## Introduction

Cardiovascular disease is the leading cause of premature morbidity and mortality in both men and women worldwide, accounting for 30.9% of global mortality and 10.3% of the global burden of disease [1,2]. Observational studies [3,4] have detected higher levels of total plasma homocysteine in patients with acute cerebrovascular disease, and elevated homocysteine levels are a risk factor for stroke [5]. It has been suggested that raised blood homocysteine concentrations should be lowered as a therapeutic approach to prevent cerebrovascular disease [6]. However, observational studies often overestimate the size of the effect and do not prove causality. 

B vitamins are used for achieving target homocysteine levels and are clearly effective at reducing the concentrations of plasma homocysteine, although their effects on cerebrovascular disease remain unclear. There are several possible reasons for these observed nonsignificant effects (1). Individual trials might have lacked the power to demonstrate a clinical benefit, especially if event rates were lower than expected, and these studies involve broad confidence intervals (2). The relationship between homocysteine levels and incident stroke was described initially by observational studies, which always overestimate the effect of this relationship (3). Most trials were conducted among patients with pre-existing disease as a secondary prevention strategy. Thus, it is possible that B-vitamin supplementation could have a greater primary protective than secondary effect (4). Lastly, the duration of follow-up could have been shorter than was needed to demonstrate a clinical benefit, or different types of supplements might have resulted in a biased view of the study question.

B-vitamin supplementation has been studied in numerous, large-scale, randomized, controlled trials [7-24] for primary and secondary prevention of cardiovascular outcomes. We could gain insight into the risk of stroke with B-vitamin supplementation and a placebo with a study involving a long-term follow-up period and proper collection of stroke data. Therefore, we conducted a systematic review and meta-analysis of pooled data from randomized controlled trials to evaluate the possible effect of B-vitamin supplementation on stroke. 

## Methods

### Data sources, search strategy, and selection criteria

This review was conducted and reported according to the Preferred Reporting Items for Systematic Reviews and Meta-Analysis (PRISMA) Statement, issued in 2009 (Checklist S1) [25]. Randomized controlled trials of B-vitamin supplementation, written in the English language, were eligible for inclusion in our meta-analysis, regardless of the publication status (published, in press, or in progress), and the effects of B-vitamin supplementation on stroke were examined. The relevant trials were identified using the following procedures:

1 Electronic searches: we searched the PubMed, Embase, and Cochrane Central Register of Controlled Trials electronic databases for articles published through April 2013 and used “homocysteine” OR “folate” OR “folic acid” OR “vitamin B12” OR “cobalamin” OR “vitamin B6” OR “pyridoxine” OR “vitamin B1” OR “thiamine” AND “randomized controlled trials” AND “clinical trials” AND “human” AND “English” as the search terms. All reference lists from reports on non-randomized controlled trials were searched manually for additional eligible studies.
2 Other sources: we searched ongoing randomized controlled trials in the metaRegister of Controlled Trials, which lists trials that are registered as completed but not yet published. Furthermore, we reviewed the bibliographies of publications for potentially relevant trials. Medical subject headings, methods, patient population, interventions, and outcome variables of these studies were used to identify relevant trials.

The literature search, data extraction, and quality assessment were undertaken independently by two authors with a standardized approach, and any disagreement between these two authors was settled by discussion with a third author (YHZ) until a consensus was reached. We restricted our meta-analysis to randomized controlled trials, which are less likely to be subject to confounding biases than observational studies. There were three study inclusion criteria. (1) The study had to be a randomized controlled trial. (2) The trial had to evaluate the effects of B-vitamin supplementation compared with a placebo. (3) The duration of the study’s follow-up period had to be at least 12 months. 

### Data collection and quality assessment

All data from eligible trials were independently abstracted, in duplicate, by 2 independent investigators with a standard protocol and reviewed by a third investigator (YHZ). Any discrepancies were resolved by group discussion, and the primary authors (YHZ) made the final decision. The recorded data variables were as follows: first author or study group’s name, publication year, study design, type of blinding, number of patients, mean age, percentage of males, patient diseases, baseline total homocysteine, intervention regimes, the duration of the follow-up period, and the number of incident strokes for each treatment group. Study quality was assessed using the Jadad score [26], which is based on the 5 following subscales: randomization (1 or 0), concealment of the treatment allocation (1 or 0), blinding (1 or 0), completeness of follow-up (1 or 0), and the use of intention-to-treat analysis (1 or 0). A “score system” (ranging from 1 to 5) was developed for quality assessment. In our study, we considered a study given a score of 4 or above to be a high-quality study. 

### Statistical analysis

We recorded the result of each randomized controlled trial as dichotomous frequency data. Individual study relative risks (RRs) and risk difference (RD) with corresponding 95% confidence intervals (CIs) were calculated from event numbers extracted from each trial before data pooling. Both fixed-effect and random-effects models were used to assess the pooled RR for B-vitamin supplementation as compared with a placebo. Although both models yielded similar findings, the results from the random-effects model presented here assume that the true underlying effect varies among included trials [27,28]. Heterogeneity of the treatment effects between studies was investigated visually by L’Abbe plots as well as statistically using the heterogeneity Q statistic [29,30]. We explored potential heterogeneity in estimates of the treatment effects with univariate meta-regression [31] (for net decrease in homocysteine, dose of folic acid, dose of vitamin B6, and dose of vitamin B12). Subsequently, subgroup analyses were conducted on the basis of man/woman ratio, stroke prevention, baseline homocysteine, net decrease in homocysteine, treatment regimens, dose of folic acid, dose of vitamin B6, dose of vitamin B12, disease status, the duration of the follow-up period, and study quality. We also performed a sensitivity analysis by removing each individual trial from the meta-analysis. Several methods were used to check for potential publication bias. Visual inspection of funnel plots for incident stroke was conducted, Egger [32] and Begg tests [33] were also used to statistically and quantitatively assess publication bias. All reported P values are two-sided, and P values <0.05 were regarded as statistically significant for all included trials. Statistical analyses were performed using STATA software (version 10.0).

## Results

We identified 13,124 articles in our initial electronic search, of which 12,847 were excluded during an initial review based on title and abstract. We retrieved the full text for the remaining 277 articles, and 18 randomized controlled trials [7-24] met the inclusion criteria (Figure S1). Table 1 summarizes the characteristics of these trials and the important baseline information of the included 57,143 individuals. The trials included in this study compared B-vitamin supplementation to a placebo for reduction of incident stroke. The follow-up period for participants ranged from 2.0 to 7.3 years, and the number of individuals included in each trial ranged from 114 to 12,064. Seven trials [7-9,11,17,18,20] included patients with chronic kidney disease. Eight trials [12-14,16,19,21,22,24] included patients with cardiovascular disease or stroke. One trial [10] included healthy individuals. One trial [15] included patients with colorectal adenomas and no previous invasive large intestine carcinoma, and 1 trial [23] included patients with esophageal dysplasia. Although the included trials scarcely reported on the key indicators of trial quality, the quality of the trials was also assessed by the Jadad score. Overall, 5 trials [7,12,13,16,21] had a Jadad score of 5. Nine trials [8,9,11,14,15,17,19,20,24] had a score of 4. Three trials [10,22,23] had a score of 3, and the remaining trial [18] had a score of 2. 

**Table 1:** Design and characteristic of trials included in our meta-analysis.

**Source**	**Publication year**	**No. of patients**	**Mean age, y**	**Percentage male (%)**	**Disease status**	**Homocysteine (umol/L)**	**Intervention**	**Follow-up (year)**	**Jadad score**
J Heinz[7]	2010	650	61	58	End-stage renal disease	29.0	2.5 mg folic acid, 25 ug vitamin B12, and 10 mg vitamin B6; 0.1 mg folic acid, 2 ug vitamin B12, and 0.5 mg vitamin B6	2.1	5
ASFAST Study Group[8]	2006	315	56	68	Chronic renal failure	27.0	15 mg folic acid; placebo	3.6	4
DIVINe Study Group[9]	2010	238	60	75	Diabetic nephropathy	15.6	2.5 mg folic acid, 25 mg vitamin B6, and 1 mg vitamin B12; placebo	3.0	4
WAFACS Study Group[10]	2008	5442	63	0	Health professionals	NG	2.5 mg of folic acid, 50 mg of vitamin B6, and 1 mg of vitamin B12; placebo	7.3	3
Veterans Affairs Site Investigators[11]	2007	2056	66	98	Chronic kidney Disease or end-stage renal disease	22.4	40 mg of folic acid, 100 mg of vitamin B6, and 2 mg of vitaminB12; placebo	3.2	4
Visp Trial Investigators[12]	2004	3680	66	63	Ischemic stroke	12.3	25 mg of vitamin B6, 0.4 mg of vitamin B12, and 2.5 mg of folic acid; 200 μg of vitamin B6, 6 μg of vitamin B12, and 20 μg of folic acid	2.0	5
SU.FOL.OM3 Collaborative Group[13]	2010	2501	61	79	myocardial infarction, unstable angina, or ischaemic stroke	12.8	Foloc acid (560 μg), vitamin B-6 (3 mg), and vitamin B-12 (20 μg)	4.7	5
SEARCH Collaborative Group [14]	2010	12064	64	83	Myocardial infarction survivors	13.5	2 mg folic acid plus 1 mg vitamin B12 daily; placebo	6.7	4
Polyp Prevention Study Group[15]	2007	1021	57	64	Colorectal adenomas and no previous invasive large intestine carcinoma	9.8	1 mg/d of folic acid daily; placebo	7.0	4
NORVIT Trial Investigators[16]	2006	3749	63	74	Acute myocardial infarction within seven days	13.1	0.8 mg of folic acid, 0.4 mg of vitamin B12, and 40 mg of vitamin B6; 0.8 mg of folic acid and 0.4 mg of vitamin B12; 40 mg of vitamin B6; or placebo	3.3	5
FAVORIT Study Group[17]	2011	4110	52	63	Kidney transplant recipients	16.4	5.0 mg folic acid, 50 mg vitamin B6, and 1.0 mg vitamin B12; 1.4 mg vitamin B6 and 0.002 mg vitamin B12	4.0	4
M Righetti[18]	2006	114	64	55	Hemodialysis	31.7	5 mg folic acid plus vitamin B1 250 mg, vitamin B6 250 mg, vitamin B12 500 ug; untreated	2.4	2
WENBIT Study Group[19]	2008	3096	62	80	Undergoing coronary angiography	NG	Folic acid, 0.8 mg, plus vitamin B12, 0.4 mg, plus vitamin B6, 40 mg; folic acid plus vitamin B12; vitaminB6 alone; placebo	3.1	4
EM Wrone[20]	2004	510	60	50	End-stage renal disease	32.9	15 mg folic acid, 12.5 mg vitamin B6, 6ug vitamin B12; 5 mg folic acid, 12.5 mg vitamin B6, 6ug vitamin B12; 1 mg folic acid, 12.5 mg vitamin B6, 6ug vitamin B12	2.0	4
VITATOPS Study Group [21]	2012	8164	63	64	Recent transient ischaemic attack or stroke	14.3	2 mg folic acid, 25 mg vitamin B6, and 0·5 mg vitamin B12; placebo	3.4	5
A Liem[22]	2005	593	65	78	Stable coronary artery disease	12.1	0.5 mg folic acid; usual care	3.2	3
The Linxian Nutrition Intervention Trial[23]	1996	3318	54	44	Esophageal dysplasia	NG	0.8 mg folic acid, 6 mg vitamin B6, and 0.018 mg vitamin B12; placebo	6.0	3
(HOPE) 2 Investigators[24]	2006	5522	69	72	Vascular disease or diabetes	12.2	2.5 mg of folic acid, 50 mg of vitamin B6, and 1 mg of vitamin B12; placebo	5.0	4

**Figure 1:**
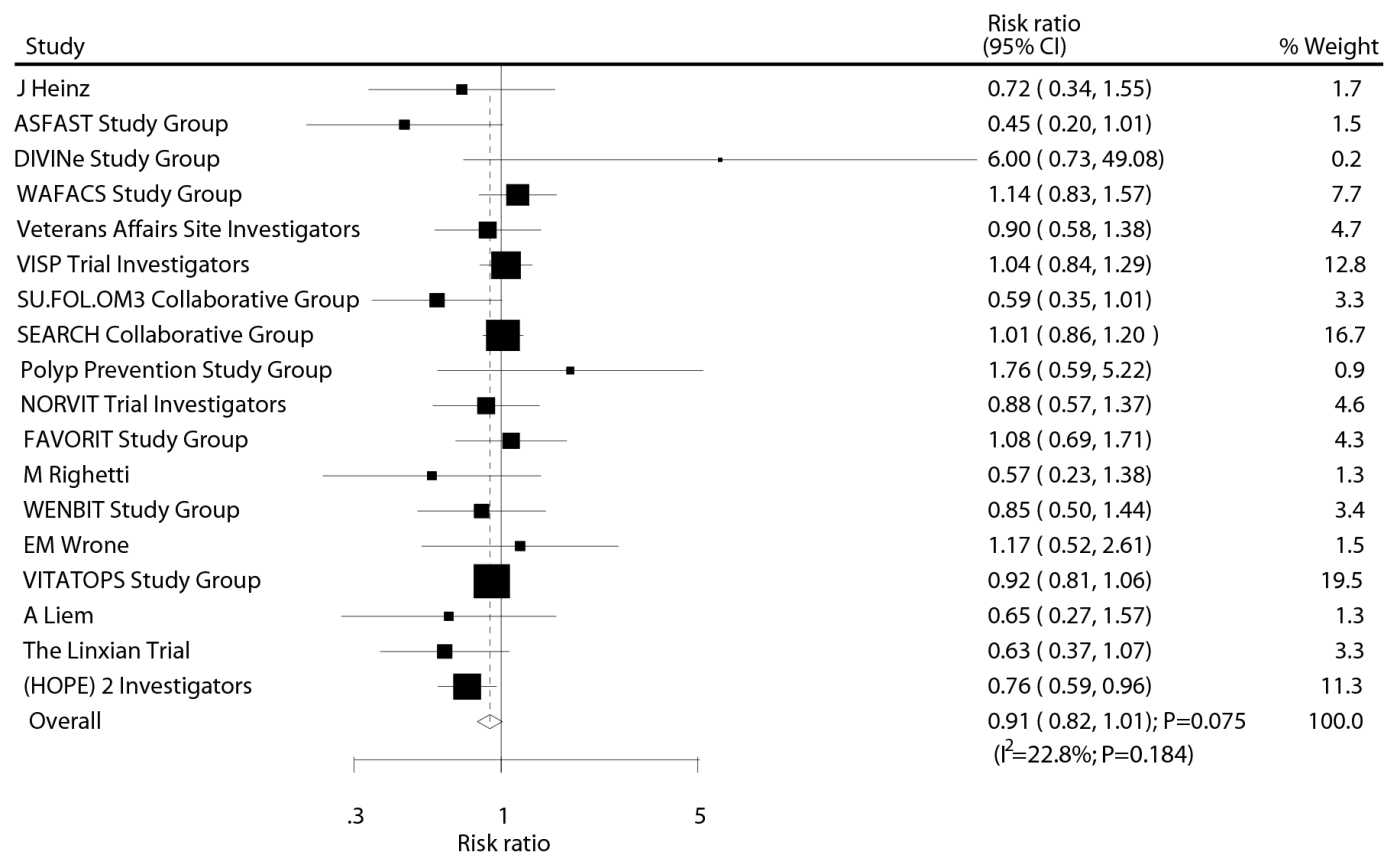
Effect of B vitamins supplementation on the risk of stroke.

**Figure 2:**
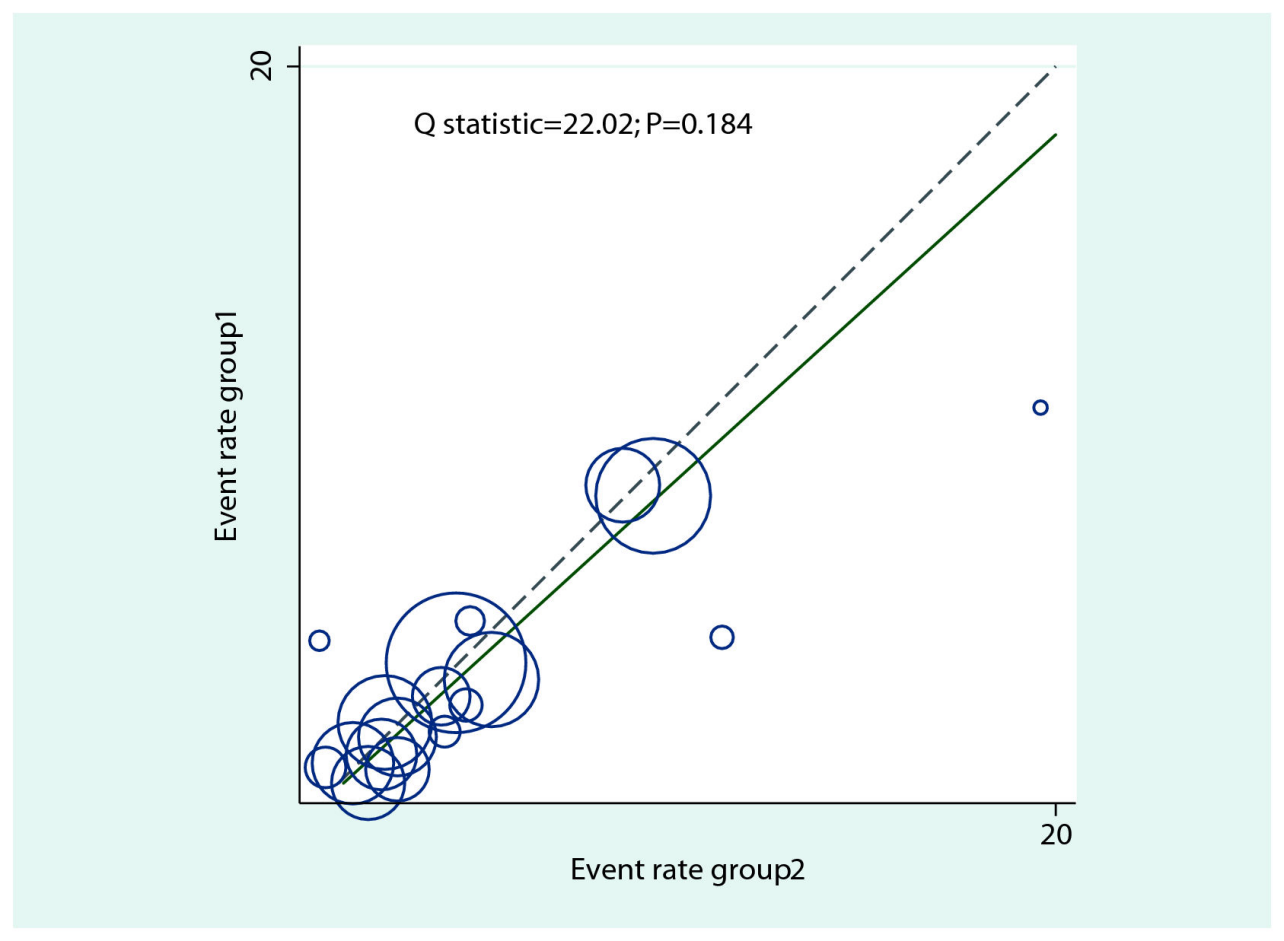
L’Abbe plots for stroke.

After pooling the included trials, we noted that B-vitamin supplementation accompanied a 9% reduction in incident stroke, but there was no evidence that B-vitamin supplementation protected against stroke risk (RR, 0.91, 95%CI: 0.82 to 1.01, P = 0.075; RD, -0.003, 95%CI: -0.007 to 0.001, P = 0.134; Figure 1). No statistically significant heterogeneity was observed between trials for incident stroke (I^2^ = 22.8%). Furthermore, the L’Abbe plots also did not display evidence of heterogeneity (Q statistic, 22.02; P = 0.184; Figure 2). 

Sensitivity analysis was conducted for incident stroke. We excluded the DIVINe trial [9], as this trial specifically included patients with a glomerular filtration rate less than 50, which may have contributed to a high mortality rate. After this exclusion, we could conclude that B-vitamin supplementation was associated with a nonsignificant reduction in the risk of stroke (RR, 0.91; 95%CI, 0.83–1.00; P = 0.06). In a cumulative meta-analysis for incident stroke, the originally proposed nonsignificant B-vitamin effect was refuted by the evidence accumulated up to 2006 (RR, 0.82; 95%CI: 0.68–0.99; P = 0.04). A continued small effect with borderline no statistical significance is observed with the 2007 and later data (Figure S2). 

Heterogeneity testing for the analysis indicated a P > 0.10 for incident stroke, and we easily concluded that heterogeneity was not significant in the overall analysis, which suggests that most variation was attributable to chance alone. Furthermore, we also conducted a meta-regression analysis [31] that included net decrease of homocysteine, dose of folic acid, dose of vitamin B6, and dose of vitamin B12. However, these variables did not appear to be important factors contributing to the association between B-vitamin supplementation and incident stroke (net decrease in homocysteine, P = 0.387; dose of folic acid, P = 0.898; dose of vitamin B6, P = 0.665; and dose of vitamin B12, P = 0.387; Figure S3). Subgroup analyses were also conducted for incident stroke to minimize heterogeneity among the included trials and evaluate the effect of B-vitamin supplementation on stroke in a specific population. We noted that B-vitamin supplementation significantly reduced the risk of stroke if included trials had a man/woman ratio of more than 2 or subjects received dose of folic acid less than 1 mg. In addition, sensitivity analyses were also conducted in subgroup analyses, we excluded the DIVINe trial [9], this trial specifically included patients with a glomerular filtration rate less than 50, we could conclude that B-vitamin supplementation was associated with a significant reduction in the risk of stroke if included trials had a man/woman ratio of more than 2, subjects received folic acid, vitamin B12, and vitamin B6 concurrently, and the follow-up period was greater than 3 years. No significant differences were identified between the effect of B vitamins and placebo, based on additional factors (Table 2). 

**Table 2:** Subgroup analysis for the effect of B vitamins supplementation on stroke.

**Group**	**RR and 95%CI**	**P value**	**Heterogeneity (%**)	**P value for heterogeneity**
**Man/woman ratio**				
>2	0.83 (0.69-1.00)	0.047	37	0.12
Excluding DIVINe Study Group[9]	0.83 (0.71-0.98)	0.03	27	0.21
<2	0.96 (0.87-1.06)	0.45	0	0.43
**Stroke Prevention**				
Primary prevention	0.91 (0.73-1.12)	0.37	27	0.19
Excluding DIVINe Study Group[9]	0.90 (0.74-1.09)	0.29	15	0.31
Secondary prevention	0.91 (0.82-1.02)	0.11	29	0.21
**Baseline homocysteine (umol/L)**				
<14	0.90 (0.77-1.06)	0.22	38	0.14
14-20	1.02 (0.73-1.43)	0.91	42	0.18
Excluding DIVINe Study Group[9]	0.94 (0.82-1.07)	0.33	0	0.51
>20	0.78 (0.58-1.05)	0.10	0	0.46
**Homocysteine lowering**				
> 20%	0.89 (0.78-1.02)	0.09	31	0.17
Excluding DIVINe Study Group[9]	0.90 (0.80-1.00)	0.052	18	0.29
<20%	0.86 (0.63-1.16)	0.33	37	0.19
**Treatment regimens**				
Folic acid plus vitamin B12 plus vitamin B6	0.90 (0.81-1.01)	0.07	17	0.27
Excluding DIVINe Study Group[9]	0.91 (0.82-0.99)	0.04	5	0.40
Folic acid plus vitamin B12	1.01 (0.86-1.20)	0.86	-	-
Folic acid only	0.75 (0.36-1.57)	0.44	49	0.14
**Folic acid (mg)**				
<1	0.74 (0.58-0.94)	0.01	0	0.73
1-3	0.95 (0.83-1.09)	0.45	43	0.08
Excluding DIVINe Study Group[9]	0.94 (0.83-1.07)	0.36	37	0.13
>3	0.95 (0.72-1.25)	0.72	0	0.59
**Vitamin B6 (mg)**				
>40	0.91 (0.73-1.12)	0.36	32	0.21
40 or less	0.90 (0.78-1.04)	0.15	18	0.28
Excluding DIVINe Study Group[9]	0.91 (0.83-1.01)	0.09	0	0.46
**Vitamins B12 (mg)**				
<0.5	0.91 (0.82-1.01)	0.07	0	0.45
1	0.99 (0.80-1.22)	0.90	53	0.07
Excluding DIVINe Study Group[9]	0.97 (0.80-1.16)	0.71	47	0.13
2	0.90 (0.58-1.38)	0.62	-	-
**Disease status**				
Renal disease	0.87 (0.63-1.18)	0.37	28	0.22
Excluding DIVINe Study Group[9]	0.86 (0.67-1.10)	0.23	1	0.41
Other	0.92 (0.83-1.02)	0.12	26	0.19
**Duration of the follow-up period (years)**				
>3	0.89 (0.79-1.01)	0.07	31	0.13
Excluding DIVINe Study Group[9]	0.89 (0.80-1.00)	0.048	24	0.20
<3	0.99 (0.82-1.21)	0.95	0	0.47
**Study quality**				
High	0.92 (0.83-1.02)	0.11	21	0.22
Excluding DIVINe Study Group[9]	0.92 (0.84-1.01)	0.08	11	0.34
Low	0.80 (0.54-1.19)	0.27	45	0.14

**Figure 3:**
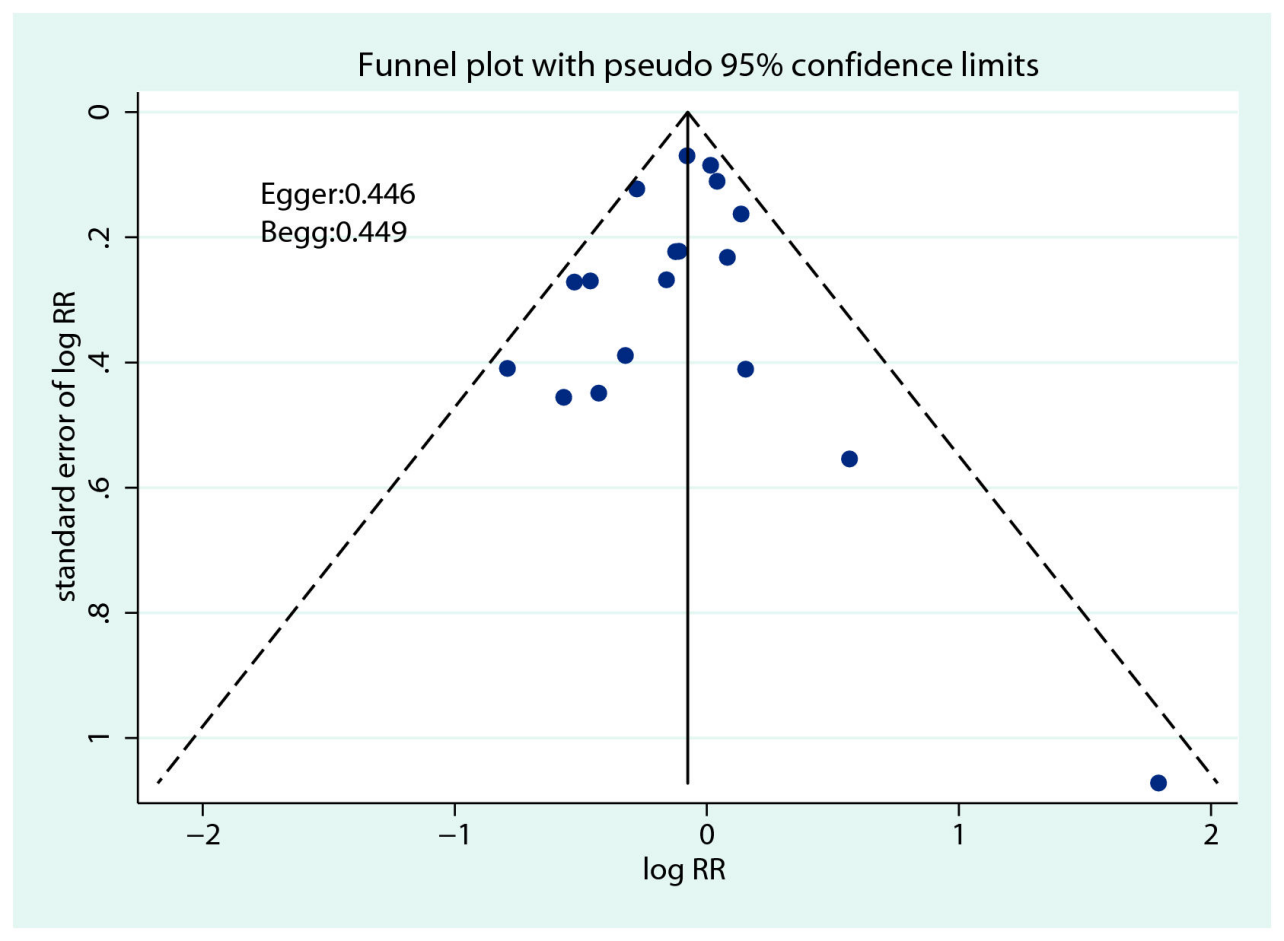
Funnel plots for stroke.

Our review of funnel plots could not rule out the potential for publication bias for stroke (Figure 3). Furthermore, Egger [32] and Begg tests [33] revealed no evidence of publication bias for stroke (P value for Egger, 0.446; P value for Begg, 0.449). 

## Discussion

Several previous observational studies [3-5] have suggested that B-vitamin supplementation has a small effect on incident stroke. Brattstrom [3] and Lindgren [4] performed case-control studies and suggested that moderate homocysteinemia might be a risk factor for arteriosclerotic cerebrovascular disease. Furthermore, Vollset and colleagues [5] performed a prospective study of plasma total homocysteine, cardiac death, and total mortality in Norway. This prospective study included 4,766 individuals aged 65–67 and found that a total homocysteine increase of 5 µmol/L was associated with a 49% increase in total mortality, a 50% increase in cardiac death, and a 104% increase in other-cause death. However, observational studies may overestimate the effect of this relationship. Our quantitative study included 57,143 individuals from 18 trials with a broad range of baseline characteristics. The results of our meta-analysis suggest that B-vitamin supplementation has no effect on the incidence of stroke. 

Our study’s findings were inconsistent with a meta-analysis [34] of stroke prevention that was published in 2007, which suggested that folic acid supplementation can effectively reduce the risk of stroke in primary prevention. In a cumulative meta-analysis of our study, B-vitamin supplementation was associated with a significant reduction in the risk of stroke by evidence accumulated up to 2006, which is consistent with the recommendations for folic acid supplementation and stroke in 2007 [34], but the significant B-vitamin effect was refuted by data gathered since 2007. Huang and colleagues [35] performed a meta-analysis indicated that B-vitamin significantly reduced the risk of stroke; however, the study did not provide the outcomes of stroke in specific subsets, and included Saposnik and colleagues’ trial [36], this trial repeatedly provided additional findings from the HOPE 2 trial [24]. Finally, Gao et al. [37] performed a meta-analysis and found that B-vitamin supplementation for homocysteine reduction play an important role on the risk of stroke events, especially in subgroups with more than 3 years follow-up time (RR, 0.92; 95%CI, 0.84-1.00), and without background of cereal folate fortification (RR, 0.91; 95%CI, 0.83-1.00) or chronic kidney disease (RR, 0.93; 95%CI, 0.86-1.01), but these differences were not found to be statistically significant. Our study had added four additional randomized controlled trials [15,18,20,22], and the findings are consistent with the findings of previous meta-analysis [37]. There are many possible reasons for this lack of significant effect (1). The use of background B-vitamin supplementation might have impaired our ability to identify a treatment effect (2). Relatively few stroke events were reported in several studies, which contributed to broad confidence intervals and restricted us from determining an intrinsic effect (3). Differences in stroke surveillance and reporting may have contributed to the differences in stroke among the trials included (4). The duration of the follow-up periods might play an important role in influencing the risk of stroke. 

Initially, observational studies suggested that reducing plasma homocysteine by 5 µmol/L would decrease vascular risk by one-third [38]; however, in a stratified analysis, our study suggested that risk of stroke reductions associated with lowering homocysteine would be much more modest. In this study, lowering homocysteine more than 20% was associated with an 11% reduction in stroke, and lowering homocysteine less than 20% provided a 14% reduction in stroke risk, but these differences were not statistically significant. These findings, when compared with the findings of previous individual randomized controlled trials, support the conclusion made by nearly all included individual trials. The WAFACS study [10] indicated that B-vitamin supplementation increased the risk of stroke by 14% in women with cardiovascular risk factors, but this difference was not statistically significant. Furthermore, the SEARCH Collaborative Study [14] also suggested that substantial long-term reduction in blood homocysteine levels with B-vitamin supplementation did not have beneficial effects on stroke, which is consistent with our stratified analysis. In addition, our meta-analysis also indicates no significant difference between the effects of B vitamins and placebo on stroke events. 

In our current meta-analysis, subgroup analyses were also performed on the basis of the factors that could affect the treatment effects [39]. These subgroups analyses suggested that the risk of stroke was significantly reduced if trials including a man/woman ratio of more than 2 or subjects received dose of folic acid less than 1 mg. When excluded the DIVINe trial [9], we concluded that B-vitamin supplementation was associated with a significant reduction in the risk of stroke if included trials had a man/woman ratio of more than 2, subjects received folic acid, vitamin B12, and vitamin B6 concurrently, and the follow-up period was greater than 3 years. The possible reasons for these could be as follows: (1) there are sex differences in the severity and treatment responsiveness of hyperhomocysteinemia, both of which are greater in men than in women [40-42]; (2) several subsets have higher stroke event rates, which increases study power to detect treatment effects. 

Our study has several limitations (1). Different types of supplements might result in a biased view of the study question (2). Several trials might not been too short to adequately identify any long-term effects of B-vitamin supplementation on stroke (3). Inherent assumptions are made for any meta-analysis. This analysis used pooled data, and individual patient data were not available, which restricted us from performing a more detailed relevant analysis and obtaining more comprehensive results. 

The findings of this study suggest that B-vitamin supplementation has no significant effects on stroke events. Subgroup analyses suggest that the risk of stroke was significantly reduced with B-vitamin supplementation compared with placebo when trials where the man/woman ratio was more than 2 were included or subjects received dose of folic acid less than 1 mg. Future studies should focus on healthy individuals with the aim of analyzing the primary prevention of stroke. We suggest that ongoing trials should be improved by ensuring that (1) any specific type of stroke and total stroke incidents should be recorded and reported normatively and be evaluated in any future trial, and (2) the role of intervention duration and dosage of supplementation should be taken into consideration before evaluating clinical outcomes. 

## Supporting Information

Checklist S1 **PRISMA Checklist.**
(DOC)

Figure S1 **Flow diagram of the literature search and trials selection process.**
(EPS)

Figure S2 **Cumulative meta-analysis of the B vitamins supplements for stroke.**
(EPS)

Figure S3 **Meta-regression of net decrease in homocyteine, dose of folic acid, dose of vitamin B6, and dose of vitamin B12 for stroke.**
(EPS)
